# Casebook: a virtual patient iPad application for teaching decision-making through the use of electronic health records

**DOI:** 10.1186/1472-6947-14-66

**Published:** 2014-08-07

**Authors:** Marcus D Bloice, Klaus-Martin Simonic, Andreas Holzinger

**Affiliations:** 1 Institute for Medical Informatics, Statistics, and Documentation, Medical University of Graz, Auenbruggerplatz 2, Graz 8036, Austria; 2 Institute of Information Systems and Computer Media, Graz University of Technology, Inffeldgasse 16c, Graz 8010, Austria

**Keywords:** Electronic health records, Decision-making, Diagnostic reasoning

## Abstract

**Background:**

Virtual Patients are a well-known and widely used form of interactive software used to simulate aspects of patient care that students are increasingly less likely to encounter during their studies. However, to take full advantage of the benefits of using Virtual Patients, students should have access to multitudes of cases. In order to promote the creation of collections of cases, a tablet application was developed which makes use of electronic health records as material for Virtual Patient cases. Because electronic health records are abundantly available on hospital information systems, this results in much material for the basis of case creation.

**Results:**

An iPad-based Virtual Patient interactive software system was developed entitled Casebook. The application has been designed to read specially formatted patient cases that have been created using electronic health records, in the form of X-ray images, electrocardiograms, lab reports, and physician notes, and present these to the medical student. These health records are organised into a timeline, and the student navigates the case while answering questions regarding the patient along the way. Each health record can also be annotated with meta-information by the case designer, such as insight into the thought processes and the decision-making rationale of the physician who originally worked with the patient. Students learn decision-making skills by observing and interacting with real patient cases in this simulated environment. This paper discusses our approach in detail.

**Conclusions:**

Our group is of the opinion that Virtual Patient cases, targeted at undergraduate students, should concern patients who exhibit prototypical symptoms of the kind students may encounter when beginning their first medical jobs. Learning theory research has shown that students learn decision-making skills best when they have access to multitudes of patient cases and it is this plurality that allows students to develop their illness scripts effectively. Casebook emphasises the use of pre-existing electronic health record data as the basis for case creation, thus, it is hoped, making it easier to produce cases in larger numbers. By creating a Virtual Patient system where cases are built from abundantly available electronic health records, collections of cases can be accumulated by institutions.

## Background

Casebook is a tablet-based, interactive Virtual Patient teaching application for undergraduate students of medicine. Within the Casebook system, the Virtual Patients are patient cases that consist of temporally organised, annotated electronic health records, such as patient presentation reports, X-ray images, lab reports, electrocardiograms, and so on. Annotations can be defined as physician notes that have been added to electronic health records for the purpose of providing some insight for the student into the physician’s decision-making rationale. These temporally organised cases are stored in specially formatted archives that can be read by the Casebook application on the iPad and displayed to the student.

The foremost aim of the Casebook Virtual Patient application is to help to teach clinical diagnostic reasoning and decision-making skills to undergraduate students of medicine through the examination of real patient cases and scenarios. The student navigates a case by traversing through a timeline of patient records, and, at strategic points throughout the case, questions must be answered by the student before they can continue. Questions can consist of the student having to make an appropriate clinical decision (the answer being revealed by the next patient record in the timeline), deciding on a working diagnosis or differential diagnosis, or for the student to answer questions regarding an inference that can be made from a lab report or X-ray image. Cases for the Casebook application are created using a text editor, by writing a descriptor file containing the case’s meta-data (questions, answers, patient record annotations) and packaging this with the patient records into a ZIP file that is read by the application. Because the cases consist of data and patient records from real-life medical situations that genuinely occurred, the cases should more closely mimic real-life scenarios than Virtual Patients that rely on produced or simulated data. We believe that Virtual Patients, even those based on real patient cases, can potentially contain misleading simulated data, especially in lab reports. Of course, real patient data can also contain badly recorded data and manually written annotations have similar potential to contain erroneous information; hence the proper vetting of cases is as always a requirement.

### Motivation for the design of Casebook

The motivation for the design and development of Casebook stems from the following observations:

● Multimedia costs can contribute to the production times of certain Virtual Patient systems, which we wished to mitigate [1-3]

● Research into how clinical reasoning and decision making is learnt suggests that access to multitudes of patient examples is key to learning decision-making skills [4-6]

● Students feel they are not prepared for clinical work: evidence shows that they feel unprepared for standard, typical patient presentations and common conditions [7-9]

Our group is of the opinion that electronic health records make for useful teaching material when formatted and packaged into coherent, logically constructed cases. Each of the three points above is discussed in more detail in the following sections.

### Reducing multimedia reliance

While most agree that the use of Virtual Patients in conjunction with traditional teaching methods is associated with large positive effects [10], depending on the type of system being developed, multimedia production can incur relatively high costs if multimedia is used extensively for Virtual Patient creation [1]. Casebook has been designed to mitigate the use of multimedia, by removing the need for producing it and relying entirely on already available electronic patient records.

Therefore, the Casebook application was designed to make use of electronic health records that are abundantly available in hospital information systems. This alleviates the need to produce rich multimedia, such as video or animation, which can contribute to the overall cost of producing individual cases on certain systems. Our group have previously reported that few Virtual Patient systems have been developed that make use of electronic health records in this way [11].

### The power of the plural

Judith Bowen’s article of 2006 on educational strategies to promote clinical reasoning is wide ranging, and covers several approaches to teaching this aspect of medicine to undergraduate students [4]. An overarching principle of her work is that experience with patients is essential in students being able to make connections between material learned and the development or ability to reason analytically. This experience builds pattern recognition, meaning the more experience a student has, the more likely they will make a correct diagnosis from the data available to them. The student must have experience with similar patients or scenarios in order to build illness scripts: the combination of conditions, symptoms, and diseases that can trigger the recall of information. This is the “power of the plural” where multiple examples facilitate transfer [5]. Other work has shown that students are found to learn best when they can calibrate themselves across multiple cases [6].

However, this experience is becoming ever harder for students to gain. Ideally students should make frequent visits to wards during their education, but due to advances in medical practice this is not the case. Because of these advances in medical science, patients are being dealt with in outpatients’ wards rather than being hospitalised, meaning students have less access to patients exhibiting common conditions, and it is these types of encounters we wish to simulate using the Casebook application [12]. The plurality required is not present in their education, and is considered to be a “major challenge”. This has resulted in students having less access to patients exhibiting common conditions, which is effecting student perceptions of preparedness, as discussed in the next section.

However, this lack of access to patients is considered to be “fertile ground” for computer simulations, where numerous cases, each of them a variation of a theme, could be displayed to students to compensate for this lack of real life experience [6,13-16].

Therefore, there is much literature that suggests the availability of multiple cases, either real patients or simulations, is advantageous for learning decision-making. The Casebook application aids the case creator by allowing for EHRs to be used as the basis for Virtual Patient cases. Its aim, therefore, is to encourage the creation of pools of cases, by allowing for case creators to make full use of the abundance of patient cases available on hospital information systems.

### Student preparedness

Work has shown that students feel unprepared for their first medical jobs upon graduation from medical school. In 2003, Goldacre et al. [7] reported on a survey conducted by them to ascertain how prepared students felt after graduation. They found that 41% of students felt unprepared for their first year of employment. According to a follow-up survey conducted six years later by Cave et al. in 2007, the situation has improved somewhat since the Goldacre survey, yet still only 59% of students agreed that they were well prepared [8]. Reasons given for this perception of unpreparedness were, for example, a lack of preparation for basic problems found on wards and a lack of emphasis on real life situations. Ochsmann et al. found similarly disconcerting results in Germany, where 60% of graduates reporting they felt unprepared [9].

In order to increase student perceptions of preparedness, multitudes of patient cases need to be accumulated – and these cases should also cover patients who are suffering from common conditions of the types that students will encounter when they first begin work as junior doctors. Casebook aids this process by allowing patient records to be used as the basis for Virtual Patient creation, thereby aiding the collection of pools of cases for students to access. Hospital information systems contain much patient data, and we believe this data has potential to be used in creating cases that deal with patients exhibiting common symptoms.

### Health record properties

Electronic health records contain a number of properties that make them desirable as teaching material. In 2000 [17] and 2005 [18], Ziv et al. described how students can reduce diagnostic error when they have been provided the opportunity to learn from their mistakes, thus making simulations ideal tools for such learning experiences. Crucially, however, they reported that students also reduce error when learning from others’ mistakes. Electronic health records provide an ideal resource for learning from error: health records, properly organised into patient cases, with the relevant annotations and discussions, provide for a unique opportunity to examine mistakes and their consequences. This would allow students to examine the cause of an error, or perhaps more importantly, the course of events that occurred which eventually led to an error. Therefore, cases do not necessarily have to present “textbook” solutions, treatments, or behaviours to encountered problems. Diagnostic error, discovered after the end of the patient’s treatment, would make for suitable cases to teach the consequences of diagnostic error [19,20].

Similarly, documentation and history-taking are important aspects of clinical skills education that would benefit from students examining both good and bad examples of medical documentation [21]. Also, colloquialisms and abbreviations are rife in medicine, and many of them are field-specific, while others still are region specific [22]. Students who study electronic health records may find that they become accustomed to such abbreviations more quickly if they encounter them in patient cases before they finish their education and begin working as doctors.

Last, electronic health records and cases based on real patients offer a unique ability to follow the patient over the course of an illness. Hirsh et al. comment that in an ideal scenario, a student should be able to observe the patient at or around the time when the initial medical decision-making is being made, and be able to follow the patient through the duration of an illness episode, even across care venues. Students should be able to witness the effects of management decisions [23]. By teaching students using cases built from patient records, they are provided the opportunity to follow the patient’s course of disease or illness, and thereby witness the effects of management decisions in real life clinical situations.

## Implementation

Taking the background research into account, our group set about designing an application that would use electronic health records, gathered from hospital information systems, to create Virtual Patient cases for use in teaching diagnostic reasoning to undergraduate students of medicine. The Casebook software itself has been developed for the Apple iPad family of devices. The tablet form factor was chosen as the software’s intended use is by small groups of four to five students within seminars, the omni-directional and easy to handle properties of the tablet being especially useful for such purposes. While our group decided to use the Apple ecosystem for the development of the Casebook application, Android tablets, such as the Google Nexus 7, feature no technical limitations that would have excluded them as a possible development platform. Software for Apple devices is written in Objective-C using the Xcode Integrated Development Environment (IDE), a freely available programming tool for Apple Macintosh computers. Our group previously reported on the development of an early version of the application both as a native, compiled application, written in Objective-C, and as an HTML5-based application that would run in a browser on Android and Apple tablets [24,25].

### Requirements

The software requirements were garnered from the theoretical background discussed in the previous sections, and consisted of three main elements. First, the software was designed to display and present Virtual Patient cases that were generated from electronic health records, organised temporally. For this purpose, a case file format was developed, which is discussed in a subsection below. In order to make it as easy as possible to generate cases, the case file format was also designed to be as simple and straightforward as possible. Second, the software must make it possible for annotations to be displayed when patient records are being viewed. Third, the software must examine the student as they navigate through the case, so that their progress can be measured and compared.

The Results section describes in detail the functionality of the software, provides screenshots, and explains how the software can be obtained.

### Case file format

The Virtual Patient cases for the Casebook application are built from electronic health records that are logically organised into a timeline, allowing the student to navigate the case and view the patient's progress as the case develops. As the case is navigated, the student must answer questions about the progression of the case (“What would you do next” questions)—the next patient record revealing the answer. Currently, only multiple choice questions are supported, however recent work has shown that interactive systems can even successfully employ natural language processing [26].

In order to create a system into which patient cases can easily be imported, a custom file format has been developed. We expect that this format will be modified further as requirements for the Virtual Patient cases develop and expand. Cases themselves are stored as standard ZIP archives. A case archive consists of two main parts: 1) a JSON (JavaScript Object Notation) descriptor file which describes the structure of the case (the order of the files and the case’s departments/sections) as well as containing all patient record annotations and multiple choice question data, and 2) the patient records themselves.

The JSON file is named case.json and contains the meta-information about the case itself. Each case has a title, a description, annotation data, questions/answers, and data regarding the structure and order of the case. The JSON descriptor file describes the order of the patient records, each of which must also be included in the ZIP archive. Patient records themselves must be in the Portable Network Graphics (PNG) format.

An example case file (Additional file 1) in the Casebook file format is provided; this file contains 15 patient records and one descriptor file. A web-based version, for viewing in any browser, is also provided in Additional file 2 as a mini-website.

There is no restriction regarding the type of electronic medical record that can be included within a case, as long as the file can be converted into the PNG format and is therefore also not restricted to any type of electronic medical record system. This would include any document-based electronic health record, and any medical image files. A table describing the time efforts required to create the example case are described in Table 1.

**Table 1:** Case creation time

**Step**	**Duration**	**Software**
***1: Medical data gathered from JMCR case report repository***	1 hour	Internet browser
***2: Medical data converted to patient record template***	2 hours	PDF viewer, word processor
***3: Case annotated by physician***	4 hours	N/A
***4: Case meta data written in JSON format (case.json)***	1 hour	Text editor, online JSON syntax validator

Details of the time expenditure for the conversion of the JMCR case for use in the Casebook application.

As Table 1 shows, the creation of this particular case was performed in just over one working day, using pre-existing material extracted from a case report on the Journal of Medical Case Reports repository [27]. These times do not take into account any operational challenges that might be expected when creating cases, such as case suitability vetting, de-identification, or case simplification: such aspects were borne by the authors of the case report itself. The operational challenges of developing cases from patient data are of course real, however, we believe them to be no more challenging than the efforts required to develop cases for classroom case-based learning scenarios.

While there is no stipulation as to who should create the cases, the application is primarily intended to be used by physicians who are involved in teaching. The physician teaching the case must not necessarily be the teacher who wrote the case. However, any annotations added to the case that contain information regarding the patient that might not be clear from simply viewing the patient records, would need to be included by the physician who dealt with the patient in question.

### Security of sensitive information

It is important to emphasise that while Casebook is an open source project, and is freely available online, patient data and cases themselves are not in any way made available or shared online by the application. Cases developed for the application are for internal teaching purposes only; the application itself is only used as a platform for displaying these cases and assessing students. Of course, teachers and institutions can share cases developed for the platform.

In terms of application security, it was also necessary to choose a platform where extremely sensitive data, such as patient data, could be stored in an encrypted form to ensure against unauthorised access. The Apple iOS range of devices support the hardware-level encryption of data using a 256-bit AES algorithm. Using enterprise management, iPads can be configured to have any number of features disabled or enforced, such as enforced use of long passwords. Other security features include auto lock (the device locks itself after a configurable amount of idle time), remote wipe (the ability to remotely delete a device’s contents), or auto wipe (the ability to stipulate that the device deletes all of its contents after a particular number of failed password attempts). When a device is erased, either remotely or because of too many failed password attempts, a technique known as crypto-shredding is used. This can delete the contents of a device in seconds by removing the decryption keys, rendering all data inaccessible. As well as this, iPads can be restricted to disable features such as the camera, or the ability to take screenshots [28]. By using such enterprise provisioning profiles, sensitive data stored on iPads can be considered secure.

In terms of de-identified patient data, the Casebook application does not enforce or restrict in any way the content that is displayed or stored on the iPad device. The development of properly de-identified and anonymised patient cases is the responsibility of those developing the cases for the software and their respective ethics committees. Of course, this means that there are operational overheads that must be considered when developing cases based on real patient health records, such as ethics commission approval and case vetting.

## Results and discussion

This section describes the functionality of the software and how it is used. A video walkthrough of Casebook in use is available in Additional file 3.

### Software functionality

**Figure 1:**
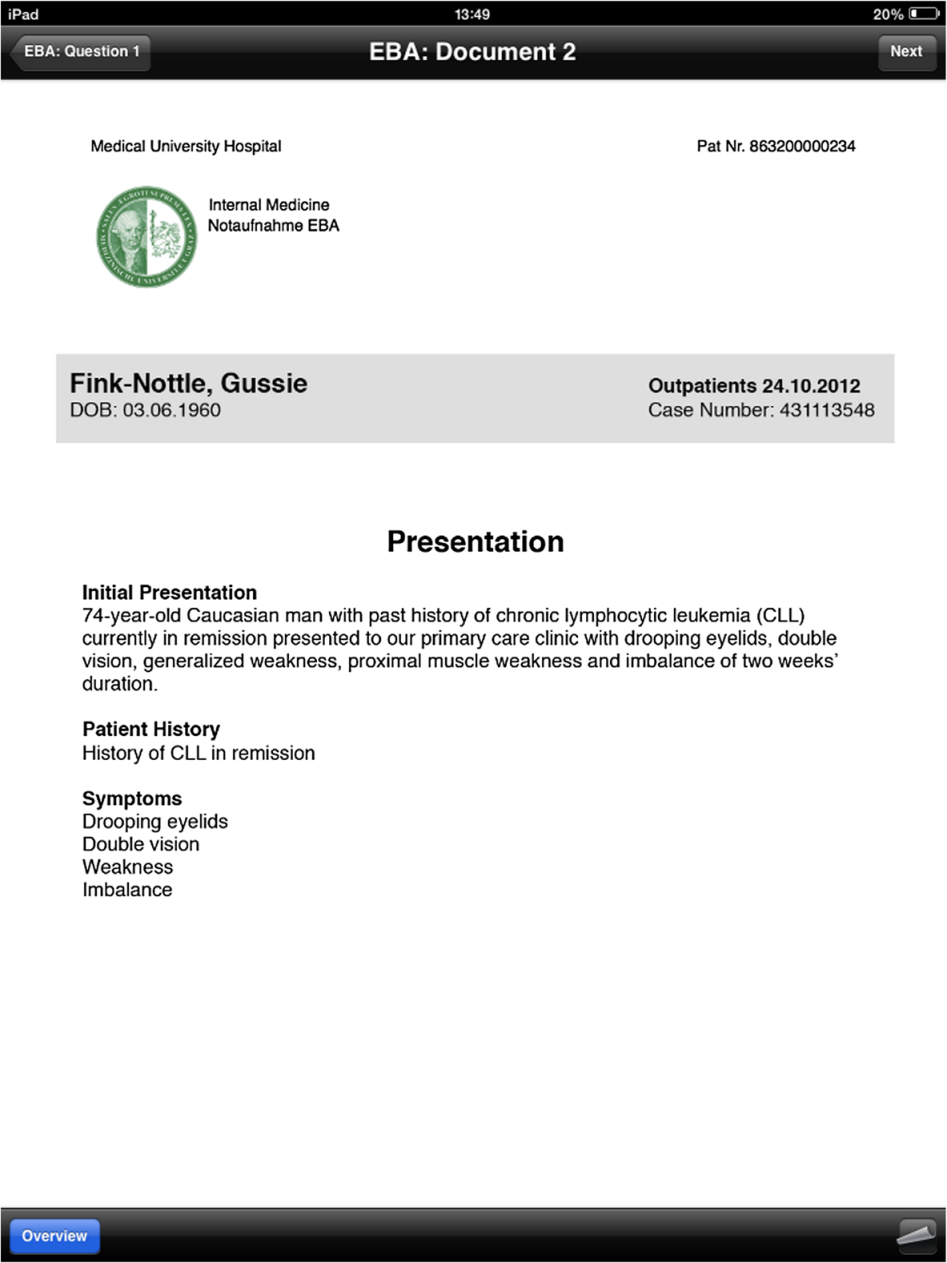
**Viewing an electronic health record.** The application’s main view is shown here, which presents to the user a health record to examine. The patient record can be scrolled and zoomed using standard pinch and zoom multi-touch gestures. By tapping ‘Overview’ the user is returned to the list of departments or sections. The user can also navigate left and right along the timeline using the navigation bar on the top of the screen. By tapping the annotations button on the bottom right, the user is presented with any notes that the teacher has provided for them, as shown in Figure 2.

**Figure 2:**
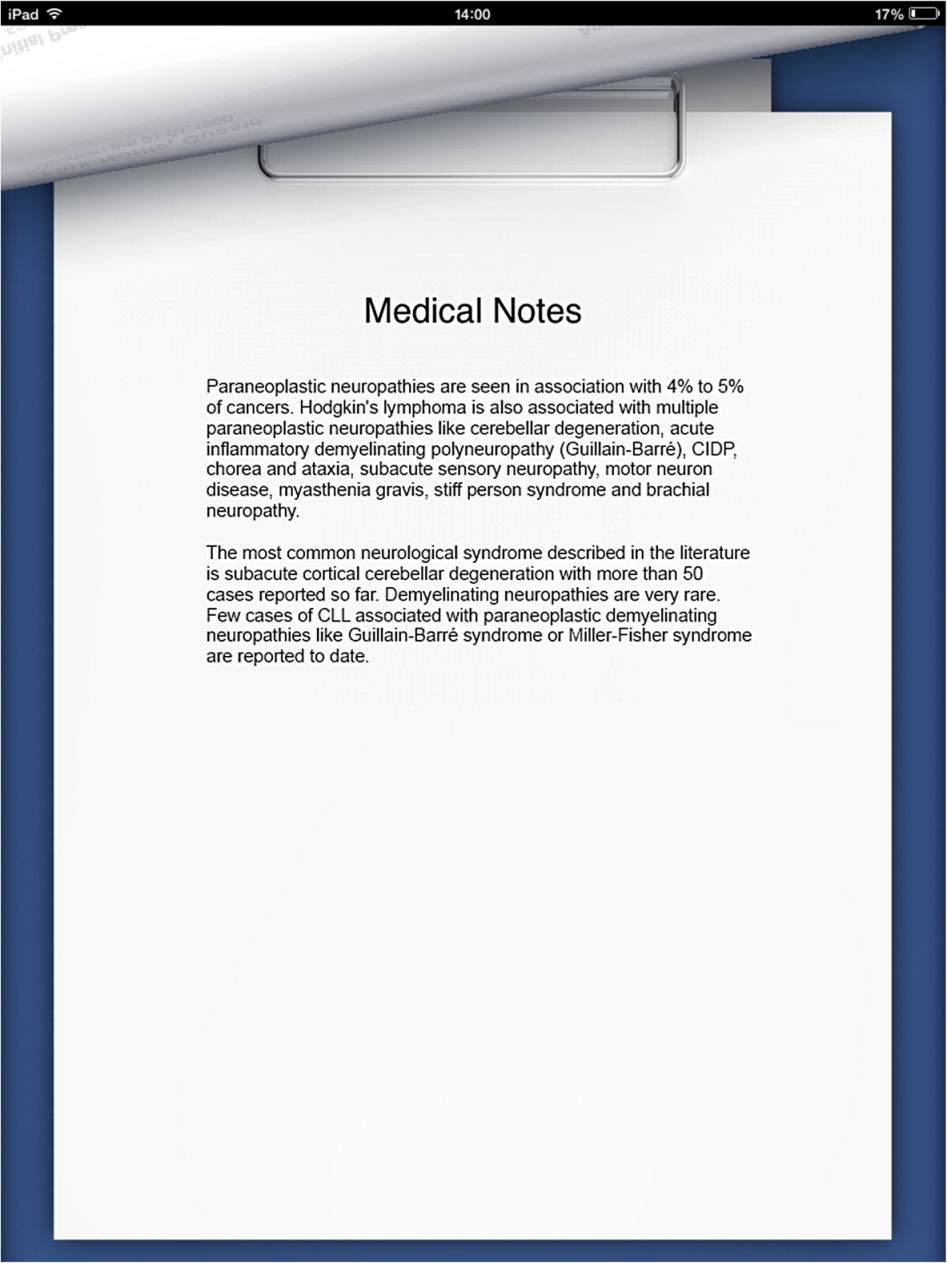
**Viewing a patient record annotation.** When viewing a patient record, students can view annotations that have been provided by the teacher or physician who created the case. Such annotations can provide insight into the decision-making processes that led the physician to perform a particular clinical action, for example.

Upon starting the application, the user is presented with an interface consisting of four tabs. The first tab contains a help screen which allows the user to get acquainted with how the application works. Users can swipe through a number of screens that describe the functionality of the application, including a description of the functionality of each of the toolbar icons (Additional file 3 contains a video of the application in use).The cases tab lists all the cases that have been imported onto the device. Once a case has been selected from the list available, the case’s sections are presented to the user. Sections are stipulated by the case creator, and should be used to split the case into logical segments to aid navigation. Once a student completes a section, the second section becomes available for selection and so on. Once a user has selected the first section, the first health record is displayed (Figure 1).As shown in Figure 1, the patient record itself dominates the centre of the screen. The top bar is the navigation bar, allowing the user to move left and right along the timeline of patient records and multiple-choice questions. The toolbar on the bottom of the screen allows the user to navigate back to the list of departments/sections (using the blue overview button) and also allows the user to view any patient record annotations that might be available. The curled up page icon on the bottom right of the screen is enabled if any annotations are available. Tapping it curls back the current patient record and presents the user with that record’s annotations (Figure 2).

As the user navigates through a patient case, they will occasionally encounter multiple-choice questions which they must answer before continuing. When a question is being presented to the user, the toolbar is slightly altered, displaying two further icons that allow the user to clear any selected answers and start over, and to submit their answers. Questions may also contain supplemental information in the form of hints, much like patient record annotations. These hints can be accessed using the icon on the toolbar, which is enabled if any hints are available. Once the user has submitted their answer, they are provided with immediate feedback and are allowed to continue to the next patient record along the timeline. Cases progress in this pattern until all sections are complete, at which point the students are given the opportunity to rate the case in terms of difficultly.

Additional file 3 contains a four minute long video walkthrough of the application in use, highlighting most aspects of the application in use.

### Learning outcomes

Currently, the Casebook application relies on multiple-choice questions to assess the student. For a purely automated assessment, multiple-choice questions are particularly suitable, yet natural language processing has also been shown to be feasible [29]. However, we emphasise and recommend that cases should be used to test the diagnostic reasoning of the student, not to test their knowledge of a particular subject. When a student answers a question, they are presented with the patient record that shows what decision the physician made in the real world, along with notes as to the diagnostic reasoning and thought processes that occurred when making this decision (in the form of annotations). It is not the intention of Casebook to assess the knowledge of the student, rather, the questions should be used to assess whether the student has a grasp of what may be the correct path to take or diagnosis to make at a particular point of the patient’s treatment. Of course, multiple-choice questions can also be used to quiz students on their knowledge, but that is not the primary aim of the Casebook project. And, as we do not want to introduce bias, we recommend that cases that are known to have involved diagnostic error should also be viewed by students [30].

### Future development

The application will continue to be further developed and will eventually include more features than those described here. The source code for the Casebook application is available under the project’s GitHub website, as seen in the Availability and requirements section at the end of this paper. Two repositories are available there: the first repository (casebookdev/Casebook-Preconfigured) is a ‘frozen’ project that contains a preconfigured build of the application including a built-in sample, fictitious, patient case. Any further development will continue in a forked, separate repository (casebookdev/Casebook). This ensures that readers can access the same application that is demonstrated in the video contained in Additional file 3. Further information on how to build and run the application can be found in the project’s GitHub wiki.

Future work will concentrate on several aspects, including minor feature enhancements as well as larger enhancements and subprojects such as the case creator. More involved enhancements will include, for example, an investigation into cases that operate in real time—that is, cases where a realistic amount of time passes between procedures. Previous work has shown the detrimental effects of time constraints on assessment, and is an area we wish to pursue [31]. In such a scenario, students would use the iPad over the course of a few days. New developments in the case appear slowly, and push notifications ensure the student is made aware of any changes in the patient's status.

Finally, cases must currently be created manually by collecting the health records required and describing their order, annotations, and question data in the case.json descriptor file. However, the procedure is rather inelegant with regard to the manual creation of the case.json descriptor file. To aid case creation, a ‘case creator’ application will be developed as a subproject. The case creator will facilitate case development through the use of a Graphical User Interface, by allowing for cases to be structured using a drag and drop interface. Annotation and question data would also be added using the GUI. The proposed case creator software will be written in Java and made available on the project’s GitHub repository. Using the Java programming language ensures cross-platform compatibility [32]. Currently, only a small number of test cases have been developed for demonstrating the application—a case creator application will hopefully encourage more cases to be developed.

## Conclusions

Research has shown that students feel they are not prepared for their first jobs after graduating from medical school. This perception of decreased preparedness can be attributed to a lack of ward work and exposure to basic clinical practice. The ability to generate working diagnoses, however, relies on experience on the ward, experience with patients, and repeated exposure to real clinical scenarios. Advances in clinical science mean that more and more patients are being treated in outpatient wards rather than being hospitalised, meaning students are even less likely to encounter patients exhibiting prototypical presentations. Virtual Patient systems have been proposed as a suitable substitute for this lack of exposure. By making it possible to quickly use cases stored in hospital information systems, we believe that the Casebook application can encourage more cases to be developed relating to prototypical situations.

Because pattern recognition is of paramount importance for the ability to generate working diagnoses and hypotheses, collections of cases are required. Therefore, pools of similar cases are required so that students can properly develop their illness scripts in order to make suitable and valid diagnoses when confronted with a new patient. The aim of the Casebook application is to emphasise the reuse of electronic health records for teaching purposes, and therefore allow for such pools to be generated.

Hence the decision to create a Virtual Patient system that makes use of the abundance of electronic health records found in hospital information systems. Our group believes there are several inherent advantages to using such an approach, including cost benefits, cases that more closely match real-life situations, the ability to create collections of similar cases, and the advantage of being able to create Virtual Patients for typical and common problems, these being the types of patients students are least likely to see during their education.

By making is possible to create cases using electronic health records, and allowing for these patient records to be annotated with physician’s notes and insights, we hope that students can gain an understanding into the thought processes and clinical reasoning of the physician who worked on the patient.

The developed application, which we have described here in detail, is one possible approach to using electronic health records for teaching decision-making and clinical reasoning. There are likely many other approaches, and indeed many other enhancements that could be made to the software to improve how it functions. It is hoped, that by making the software open-source and freely available to download, modify, and distribute under the terms of the GPL licence, other institutions may also wish to become involved in the project. The source has therefore been made available on GitHub and will continue to be hosted there as development continues.

## Availability and requirements

The following outlines the requirements and availability of the Casebook project:

● Project name: Casebook

● Project homepage: https://github.com/casebookdev

● Operating System(s): iOS

● Programming language: Objective-C

● Other requirements: iPad running iOS 6, Macintosh computer and Xcode for building project source.

● Licence: GNU GPL v2

## Competing interests

The authors declare that they have no competing interests.

## Authors’ contributions

The project was conceived by MDB, KMS, and AH. The Casebook application was developed by MDB. The final paper was read and approved by all authors.

## Pre-publication history

The pre-publication history for this paper can be accessed here:

http://www.biomedcentral.com/1472-6947/14/66/prepub

## Supplementary Material

Additional file 1 **Example case file.** This file contains an example case in ZIP format that consists of 15 health records and a single JSON descriptor file (case.json) in the Casebook file format. The descriptor file describes the order of the health records, contains any annotations for each of the health records, and contains any questions that appear between health records. Portions of this case, including text, were extracted from a case published in the Journal of Medical Case Reports, an open-access journal where articles are made available under the terms of the Creative Commons Attribution Licence [27]. Click here for file

Additional file 2 **Mini-website.** HTML version of the example case that can be viewed using a browser. See Additional file 1 for this case in the Casebook file format. Click here for file

Additional file 3 **Video walkthrough of the Casebook application in use.** This video (QuickTime format, 4m00s in duration) contains a walkthrough of the Casebook application in use. In the walkthrough, a short example case is chosen from the list of available cases, and is navigated through until the end of the first section of the case. Click here for file

## References

[B1] Huang G Reynolds R Candler C Virtual patient simulation at US and Canadian medical schools Acad Med 2007 82 5 446 451 17457063 10.1097/ACM.0b013e31803e8a0a

[B2] Ellaway R Poulton T Fors U McGee J Albright S Building a virtual patient commons Med Teach 2008 30 2 170 174 18464142 10.1080/01421590701874074

[B3] Salminen H Zary N Björklund K Toth-Pal E Leanderson C Virtual patients in primary care: developing a reusable model that fosters reflective practice and clinical reasoning J Med Internet Res 2014 16 1 e3 24394603 10.2196/jmir.2616 PMC3906652

[B4] Bowen J Educational strategies to promote clinical diagnostic reasoning N Engl J Med 2006 355 21 2217 2225 17124019 10.1056/NEJMra054782

[B5] Norman G Dore K Krebs J Neville A The power of the plural: effect of conceptual analogies on successful transfer Acad Med 2007 82 10 Suppl S16 S18 17895680 10.1097/ACM.0b013e3181405ad7

[B6] Bordage G Why did I miss the diagnosis? Some cognitive explanations and educational implications Acad Med 1999 74 10 Suppl S138 S143 10536619 10.1097/00001888-199910000-00065

[B7] Goldacre M Lambert T Evans J Turner G Preregistration house officers' views on whether their experience at medical school prepared them well for their jobs: national questionnaire survey BMJ 2003 326 7397 1011 1012 12742922 10.1136/bmj.326.7397.1011 PMC154758

[B8] Cave J Goldacre M Lambert T Woolf K Jones A Dacre J Newly qualified doctors' views about whether their medical school had trained them well: questionnaire surveys BMC Med Educ 2007 7 38 17945007 10.1186/1472-6920-7-38 PMC2203980

[B9] Ochsmann E Zier U Drexler H Schmid K Well prepared for work? Junior doctors' self-assessment after medical education BMC Med Educ 2011 11 99 22114989 10.1186/1472-6920-11-99 PMC3267657

[B10] Cook DA Erwin PJ Triola MM Computerized virtual patients in health professions education: a systematic review and meta-analysis Acad Med 2010 85 10 1589 1602 20703150 10.1097/ACM.0b013e3181edfe13

[B11] Bloice MD Simonic KM Holzinger H On the usage of health records for the design of virtual patients: a systematic review BMC Med Inform Decis Mak 2013 13 103 24011027 10.1186/1472-6947-13-103 PMC3846661

[B12] Whitcomb M Ambulatory-based clinical education: Flexner revisited Acad Med 2006 81 2 105 106 16436569 10.1097/00001888-200602000-00001

[B13] Holzinger A Kickmeier-Rust MD Wassertheurer S Hessinger M Learning performance with interactive simulations in medical education: Lessons learned from results of learning complex physiological models with the HAEMOdynamics SIMulator Comput Educ 2009 52 2 292 301

[B14] Holzinger A Emberger W Wassertheurer S Neal L Design, development and evaluation of online interactive simulation software for learning human genetics Elektr Infor (e&i) 2008 125 5 190 196

[B15] Zary N Hege I Heid J Woodham L Donkers J Kononowicz AA Adlassnig KP, Mantas J, Masic I Enabling Interoperability, Accessibility and Reusability of Virtual Patients Across Europe - Design and Implementation Proceedings of the twenty-second International Conference on Medical Informatics Europe: 30 August – 2 September 2009 2009 Sarajevo: Ios Press 826 830 19745428

[B16] eViP Electronic Virtual Patients http://www.virtualpatients.eu

[B17] Ziv A Small S Wolpe P Patient safety and simulation-based medical education Med Teach 2000 22 5 489 495 21271963 10.1080/01421590050110777

[B18] Ziv A Ben-David S Ziv M Simulation based medical education: an opportunity to learn from errors Med Teach 2005 27 3 193 199 16011941 10.1080/01421590500126718

[B19] Britton M Diagostic errors discovered at autopsy Acta Med Scand 1974 196 1–6 203 210 4422553 10.1111/j.0954-6820.1974.tb00996.x

[B20] Winters B Custer J Galvagno SM Colantuoni E Kapoor SG Lee HW Goode V Robinson K Nakhasi A Pronovost P Newman-Toker D Diagnostic errors in the intensive care unit: a systematic review of autopsy studies BMJ Qual Saf 2012 21 894 902 10.1136/bmjqs-2012-000803 22822241

[B21] Rouf E Chumley HS Dobbie AE Electronic health records in outpatient clinics: perspectives of third year medical students BMC Med Educ 2008 8 13 18373880 10.1186/1472-6920-8-13 PMC2294117

[B22] Lee P Hunter TB Taljanovic M Musculoskeletal colloquialisms: how did we come up with these names? Radiographics 2004 24 4 1009 1027 15256625 10.1148/rg.244045015

[B23] Hirsh D Ogur B Thibault G Cox M “Continuity” as an organizing principle for clinical education reform N Engl J Med 2007 356 8 858 866 17314348 10.1056/NEJMsb061660

[B24] Bloice MD Simonic KM Kreuzthaler M Holzinger A Development of an interactive application for learning medical procedures and clinical decision making In Information Quality in e-Health 2011 Berlin, Heidelberg: Springer Berlin Heidelberg 211 224

[B25] Holzinger A Treitler P Slany W Making Apps Useable on Multiple Different Mobile Platforms: On Interoperability for Business Application Development on Smartphones Multidisciplinary Research and Practice for Information Systems, Lecture Notes in Computer Science, LNCS 7465 2012 176 189

[B26] Oliven A Nave R Gilad D Barch A Implementation of a web-based interactive virtual patient case simulation as a training and assessment tool for medical students Stud Health Technol Inform 2011 169 233 237 21893748

[B27] Ammannagari N Chikoti S Bravin E Hodgkin's lymphoma presenting as a complex paraneoplastic neurological syndrome: a case report J Med Case Rep 2013 7 96 23566362 10.1186/1752-1947-7-96 PMC3637286

[B28] Jaquith A Apple’s iPhone and iPad: secure enough for business? Forrester Research 2010 URL: http://www.forrester.com/Apples+iPhone+And+iPad+Secure+Enough+For+Business/fulltext/-/E-RES57240?objectid=RES57240

[B29] Nirenburg S McShane M Beale S Jarrell B Fantry G Integrating cognitive simulation into the maryland virtual patient Stud Health Technol Inform 2009 142 17 224 229 19377155

[B30] Eva K What every teacher needs to know about clinical reasoning Med Educ 2005 39 1 98 106 15612906 10.1111/j.1365-2929.2004.01972.x

[B31] Gunning WT Fors UGH Virtual patients for assessment of medical student ability to integrate clinical and laboratory data to develop differential diagnoses: comparison of results of exams with/without time constraints Med Teach 2012 34 4 e222 e228 22455713 10.3109/0142159X.2012.642830

[B32] Bloice MD Wotawa F Holzinger A Luzar-Stiffler V, Dobric VH, Bekic Z Java’s Alternatives and the Limitations of Java when Writing Cross-Platform Applications for Mobile Devices in the Medical Domain ITI 2009 31st International Conference on Information Technology Interfaces: June 22-25, 2009 2009 Cavtat (Croatia): IEEE 47 54

