# Supplementary figures and images for: Casebook: a virtual patient iPad application for teaching decision-making through the use of electronic health records
Source: BMC Med Inform Decis Mak. 2014 Aug 7;14:66. doi: 10.1186/1472-6947-14-66 (PMC4149039; doi:10.1186/1472-6947-14-66)

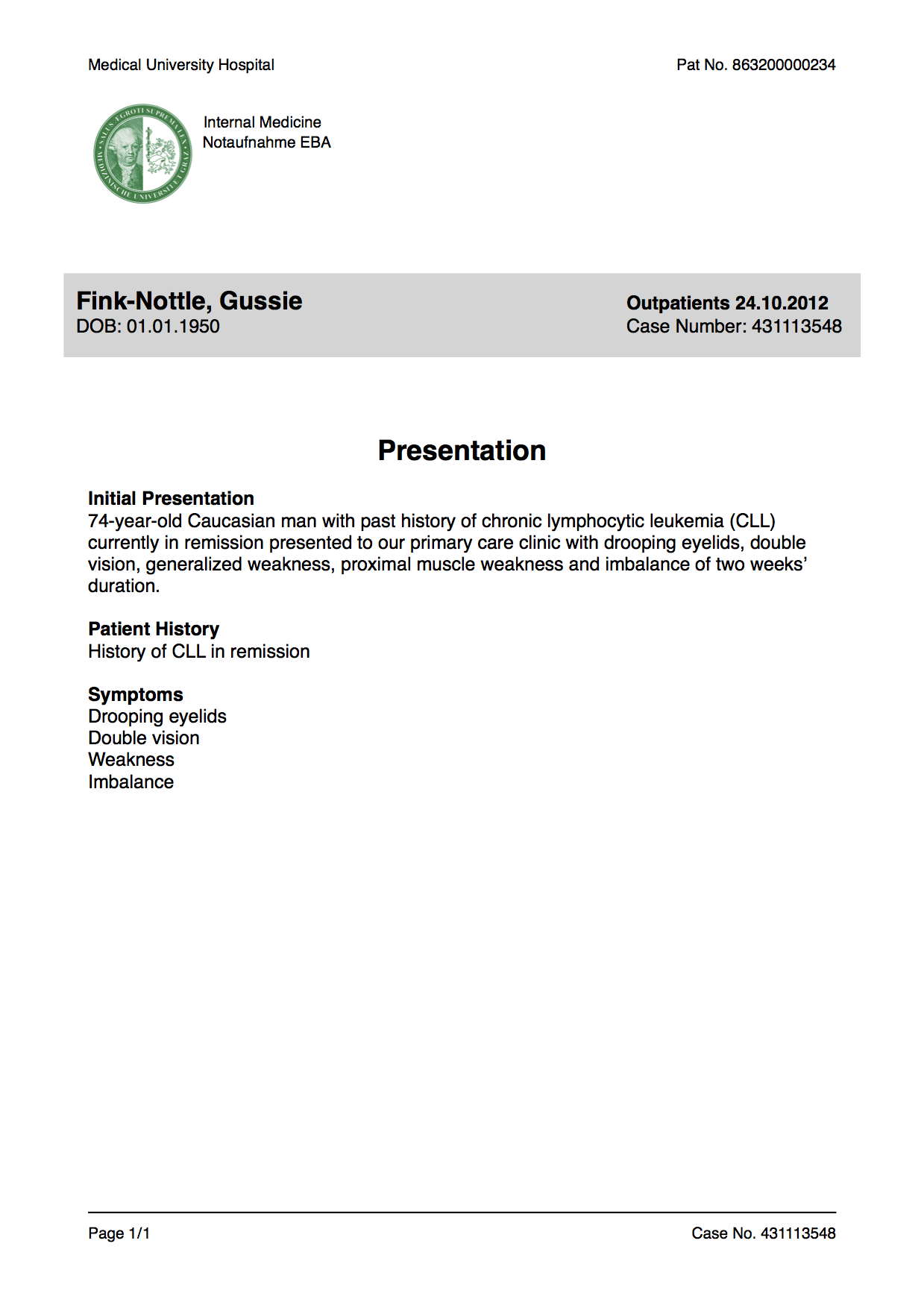

Supplement: Additional file 1 — Example case file. This file contains an example case in ZIP format that consists of 15 health records and a single JSON descriptor file (case.json) in the Casebook file format. The descriptor file describes the order of the health records, contains any annotations for each of the health records, and contains any questions that appear between health records. Portions of this case, including text, were extracted from a case published in the Journal of Medical Case Reports, an open-access journal where articles are made available under the terms of the Creative Commons Attribution Licence [27]. [file 1472-6947-14-66-S1.zip › 1.png]

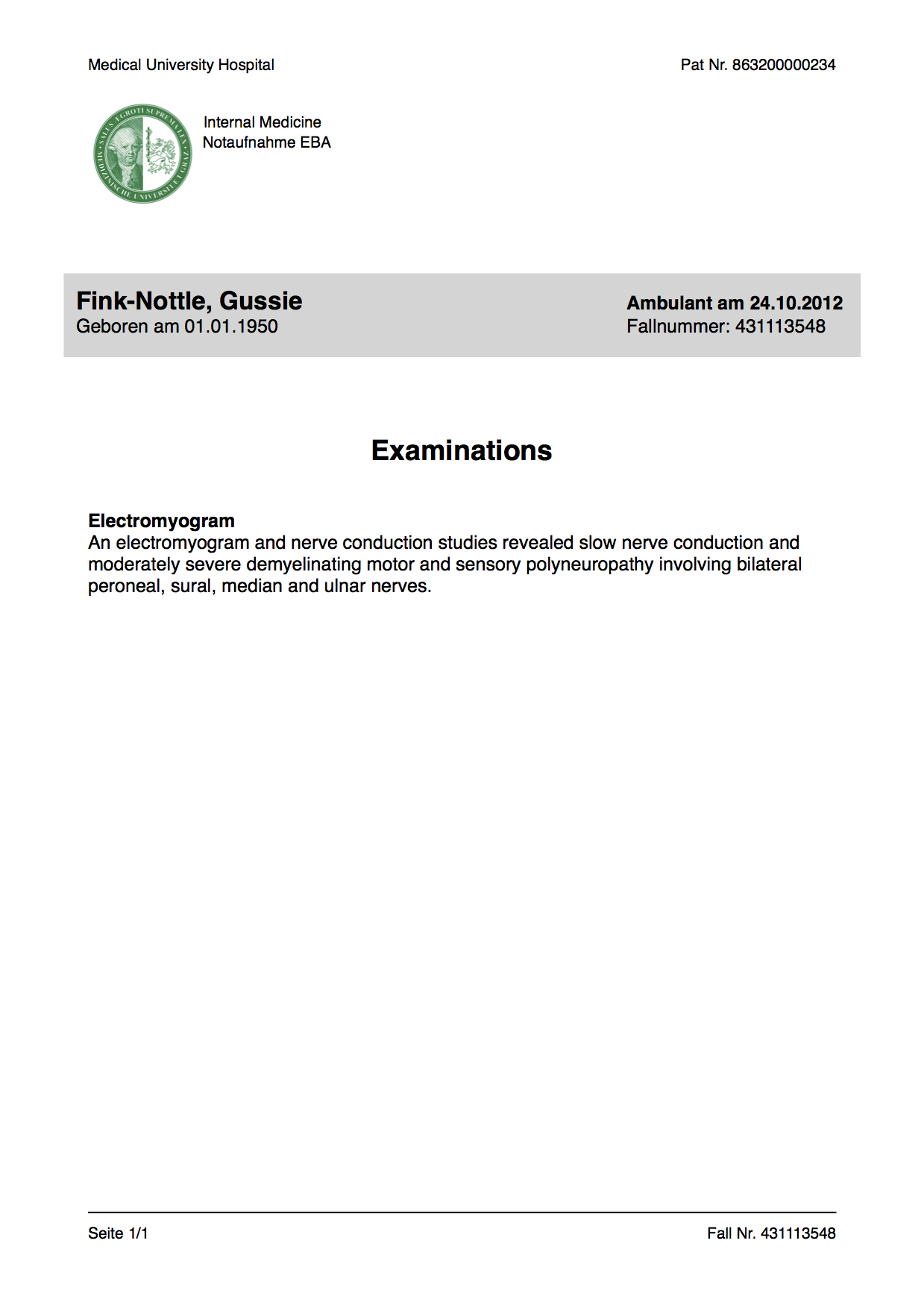

Supplement: Additional file 1 — Example case file. This file contains an example case in ZIP format that consists of 15 health records and a single JSON descriptor file (case.json) in the Casebook file format. The descriptor file describes the order of the health records, contains any annotations for each of the health records, and contains any questions that appear between health records. Portions of this case, including text, were extracted from a case published in the Journal of Medical Case Reports, an open-access journal where articles are made available under the terms of the Creative Commons Attribution Licence [27]. [file 1472-6947-14-66-S1.zip › 10.png]

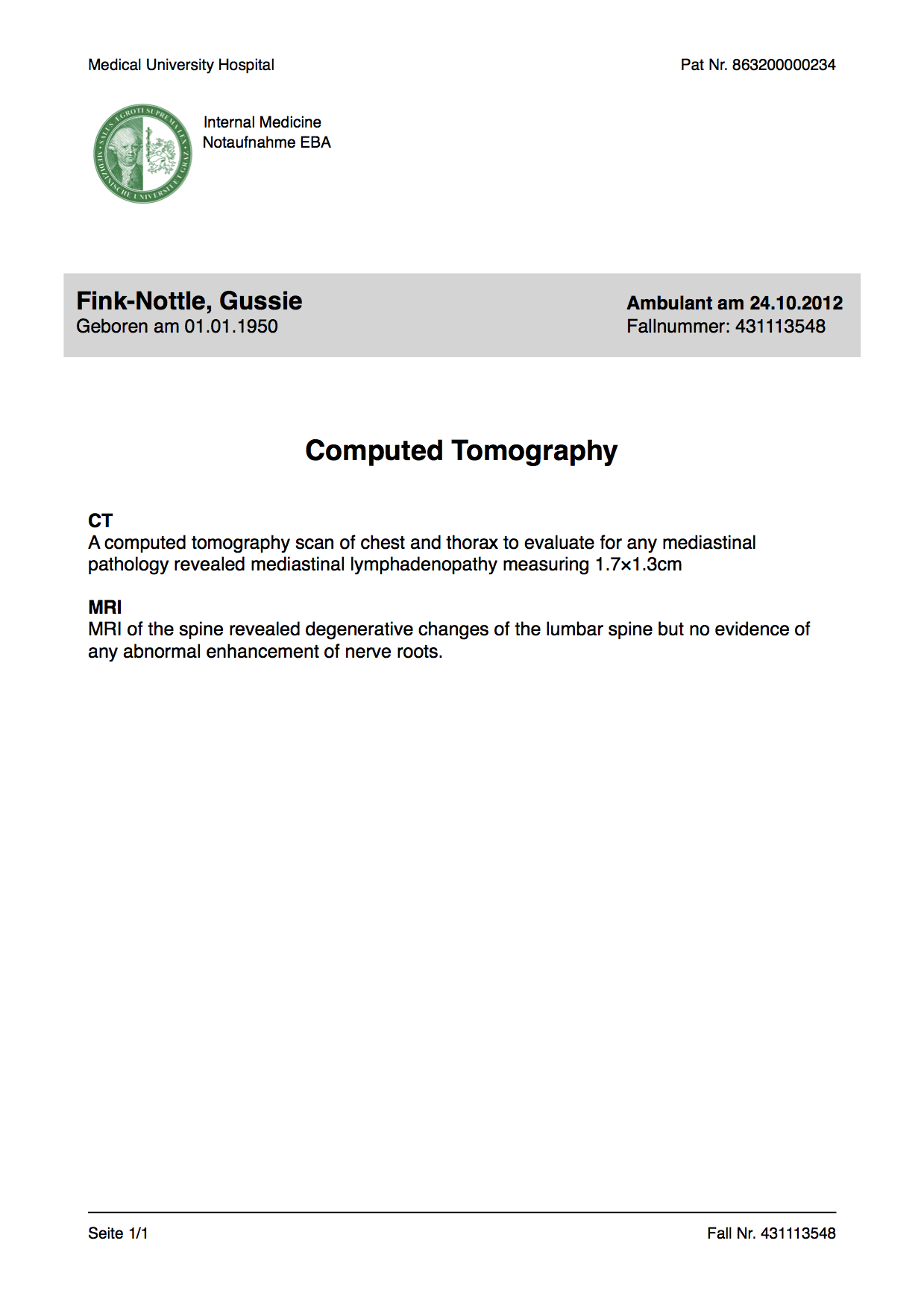

Supplement: Additional file 1 — Example case file. This file contains an example case in ZIP format that consists of 15 health records and a single JSON descriptor file (case.json) in the Casebook file format. The descriptor file describes the order of the health records, contains any annotations for each of the health records, and contains any questions that appear between health records. Portions of this case, including text, were extracted from a case published in the Journal of Medical Case Reports, an open-access journal where articles are made available under the terms of the Creative Commons Attribution Licence [27]. [file 1472-6947-14-66-S1.zip › 11.png]

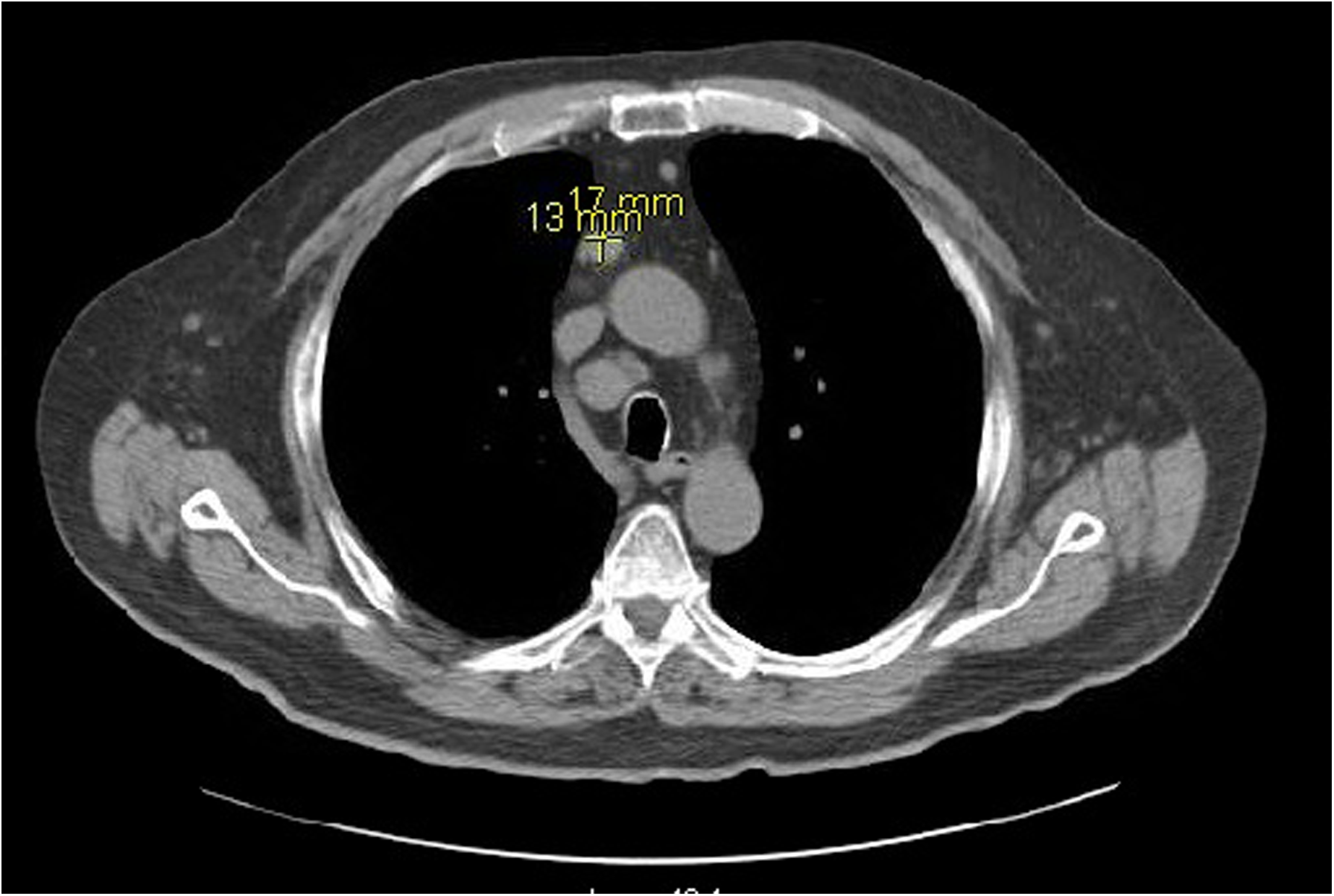

Supplement: Additional file 1 — Example case file. This file contains an example case in ZIP format that consists of 15 health records and a single JSON descriptor file (case.json) in the Casebook file format. The descriptor file describes the order of the health records, contains any annotations for each of the health records, and contains any questions that appear between health records. Portions of this case, including text, were extracted from a case published in the Journal of Medical Case Reports, an open-access journal where articles are made available under the terms of the Creative Commons Attribution Licence [27]. [file 1472-6947-14-66-S1.zip › 12.png]

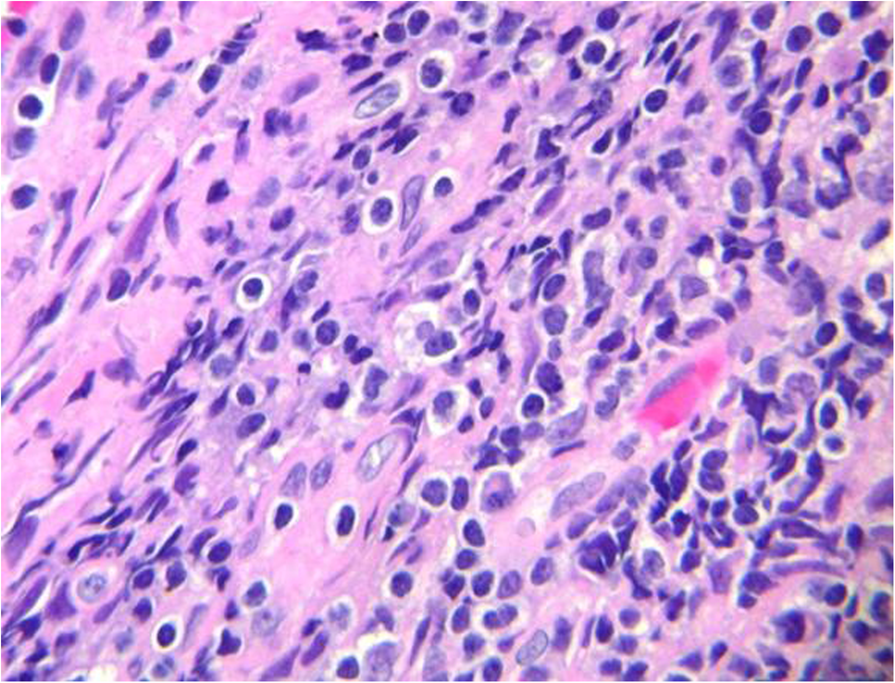

Supplement: Additional file 1 — Example case file. This file contains an example case in ZIP format that consists of 15 health records and a single JSON descriptor file (case.json) in the Casebook file format. The descriptor file describes the order of the health records, contains any annotations for each of the health records, and contains any questions that appear between health records. Portions of this case, including text, were extracted from a case published in the Journal of Medical Case Reports, an open-access journal where articles are made available under the terms of the Creative Commons Attribution Licence [27]. [file 1472-6947-14-66-S1.zip › 13.png]

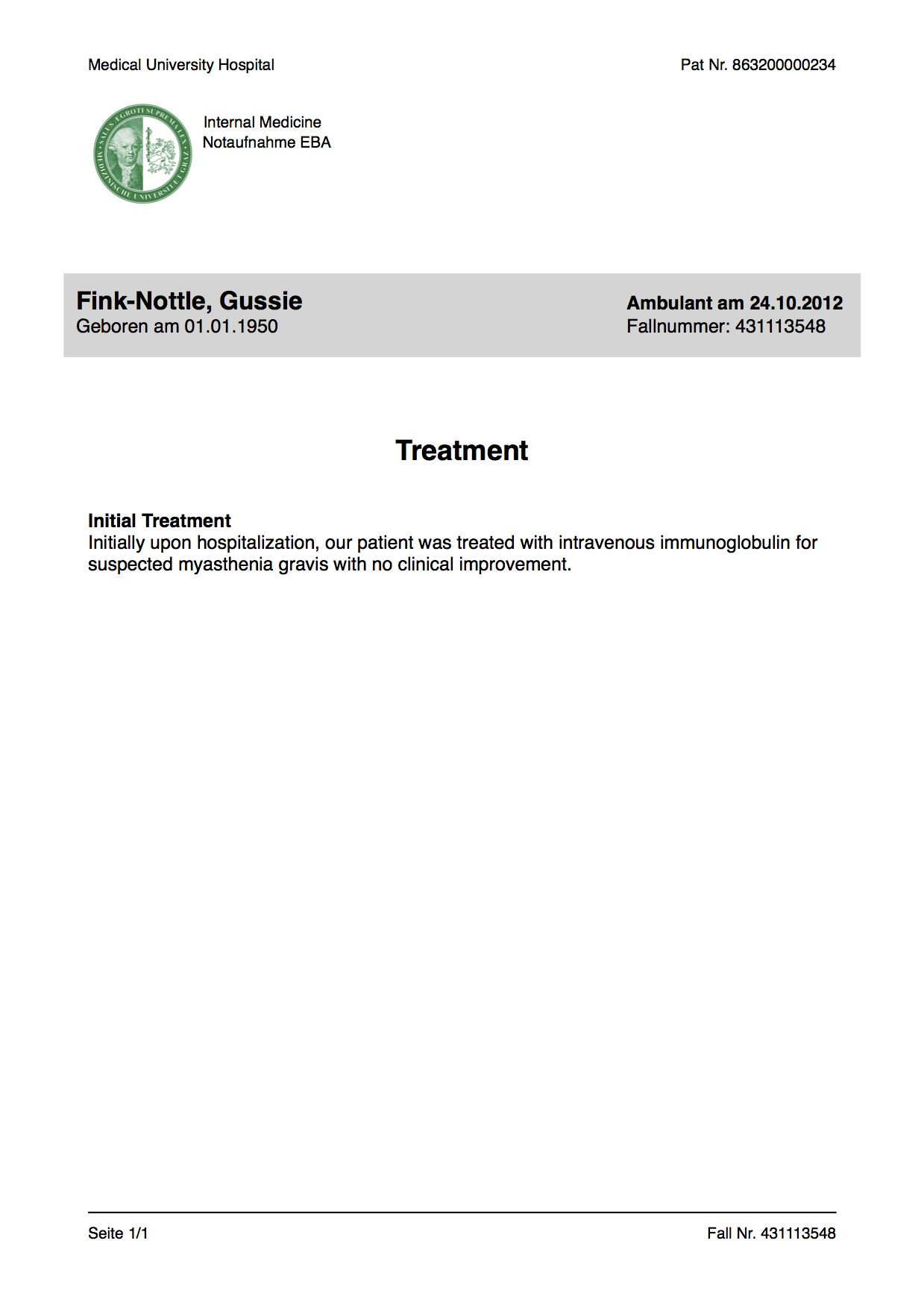

Supplement: Additional file 1 — Example case file. This file contains an example case in ZIP format that consists of 15 health records and a single JSON descriptor file (case.json) in the Casebook file format. The descriptor file describes the order of the health records, contains any annotations for each of the health records, and contains any questions that appear between health records. Portions of this case, including text, were extracted from a case published in the Journal of Medical Case Reports, an open-access journal where articles are made available under the terms of the Creative Commons Attribution Licence [27]. [file 1472-6947-14-66-S1.zip › 14.png]

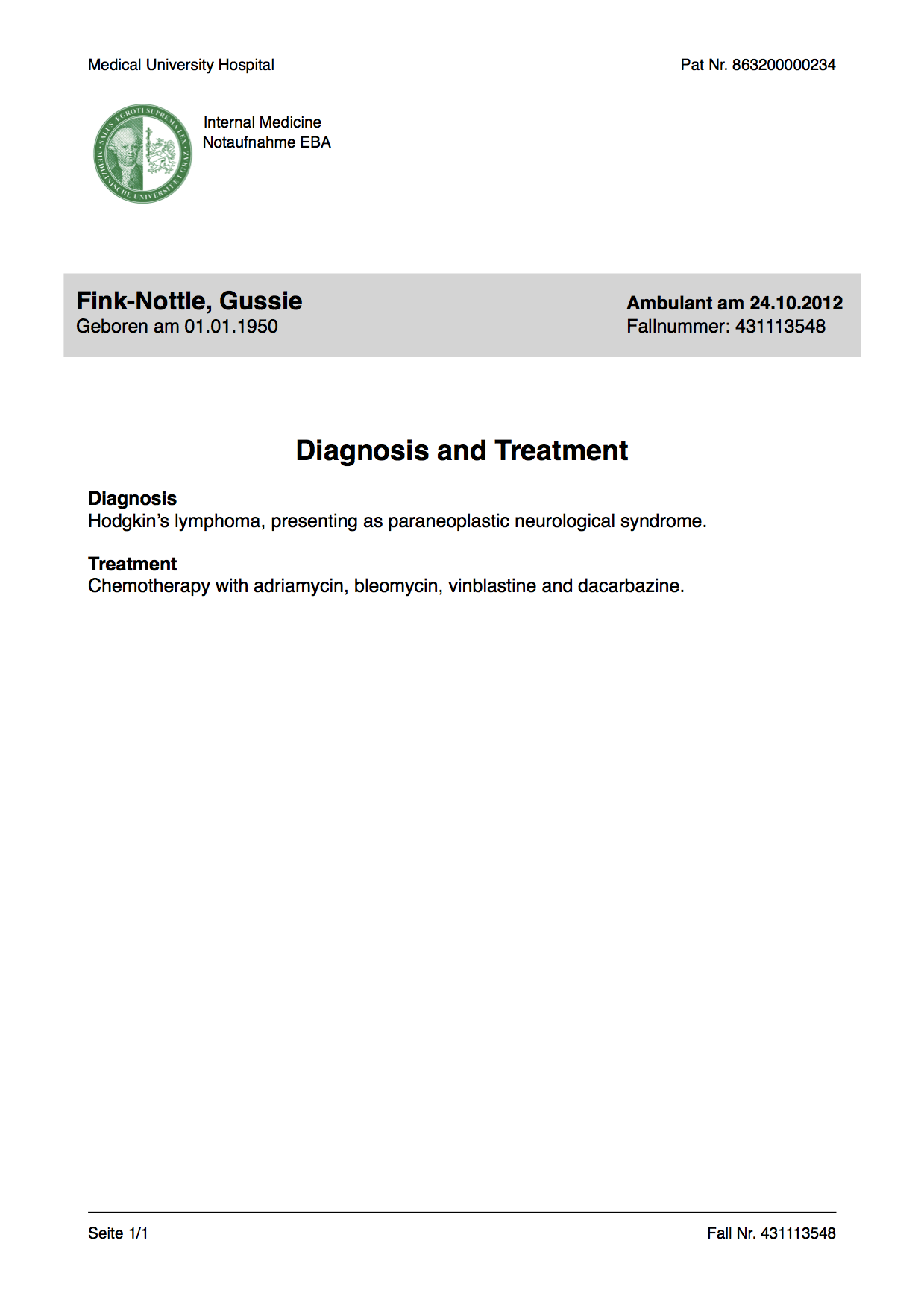

Supplement: Additional file 1 — Example case file. This file contains an example case in ZIP format that consists of 15 health records and a single JSON descriptor file (case.json) in the Casebook file format. The descriptor file describes the order of the health records, contains any annotations for each of the health records, and contains any questions that appear between health records. Portions of this case, including text, were extracted from a case published in the Journal of Medical Case Reports, an open-access journal where articles are made available under the terms of the Creative Commons Attribution Licence [27]. [file 1472-6947-14-66-S1.zip › 15.png]

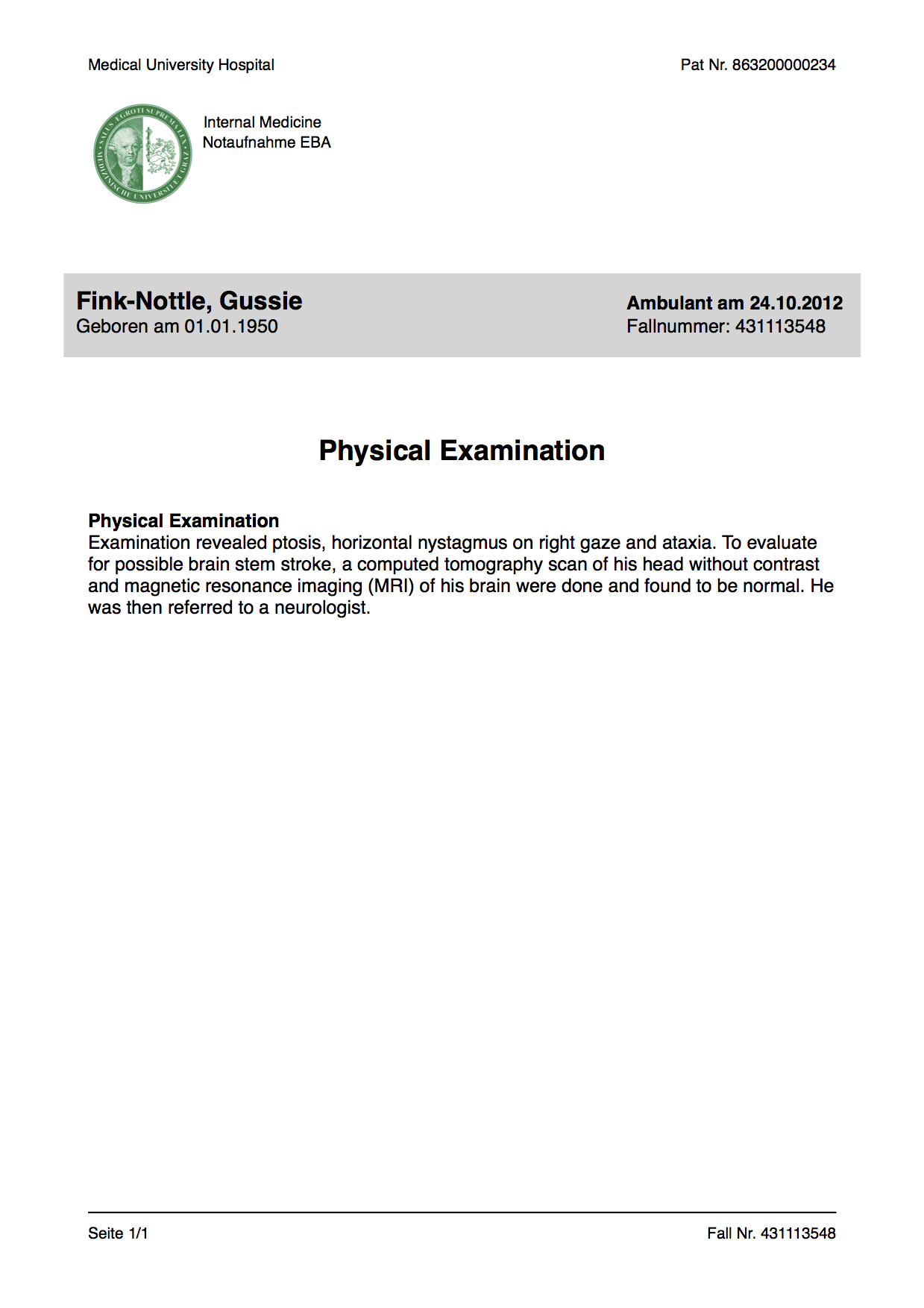

Supplement: Additional file 1 — Example case file. This file contains an example case in ZIP format that consists of 15 health records and a single JSON descriptor file (case.json) in the Casebook file format. The descriptor file describes the order of the health records, contains any annotations for each of the health records, and contains any questions that appear between health records. Portions of this case, including text, were extracted from a case published in the Journal of Medical Case Reports, an open-access journal where articles are made available under the terms of the Creative Commons Attribution Licence [27]. [file 1472-6947-14-66-S1.zip › 2.png]

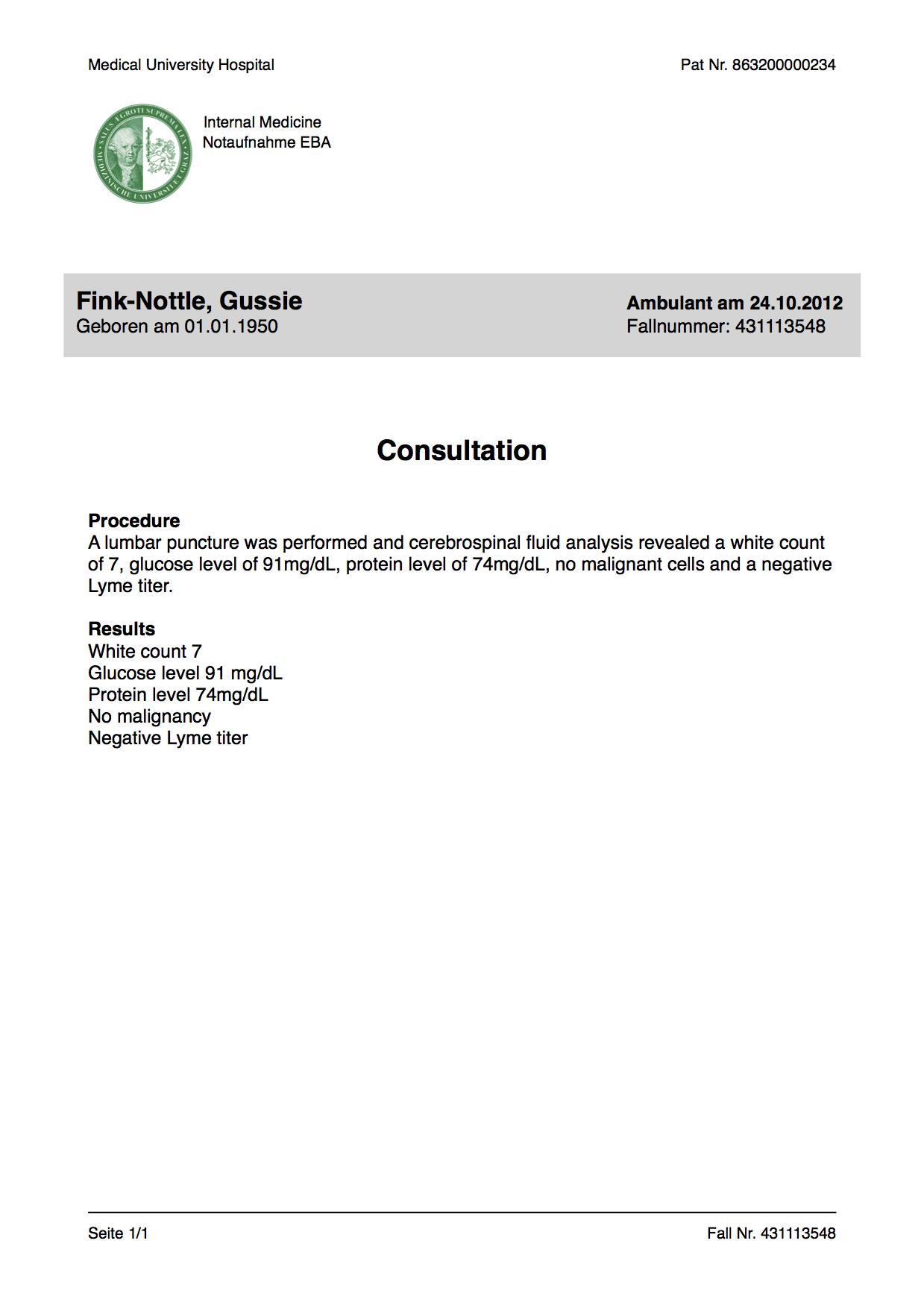

Supplement: Additional file 1 — Example case file. This file contains an example case in ZIP format that consists of 15 health records and a single JSON descriptor file (case.json) in the Casebook file format. The descriptor file describes the order of the health records, contains any annotations for each of the health records, and contains any questions that appear between health records. Portions of this case, including text, were extracted from a case published in the Journal of Medical Case Reports, an open-access journal where articles are made available under the terms of the Creative Commons Attribution Licence [27]. [file 1472-6947-14-66-S1.zip › 3.png]

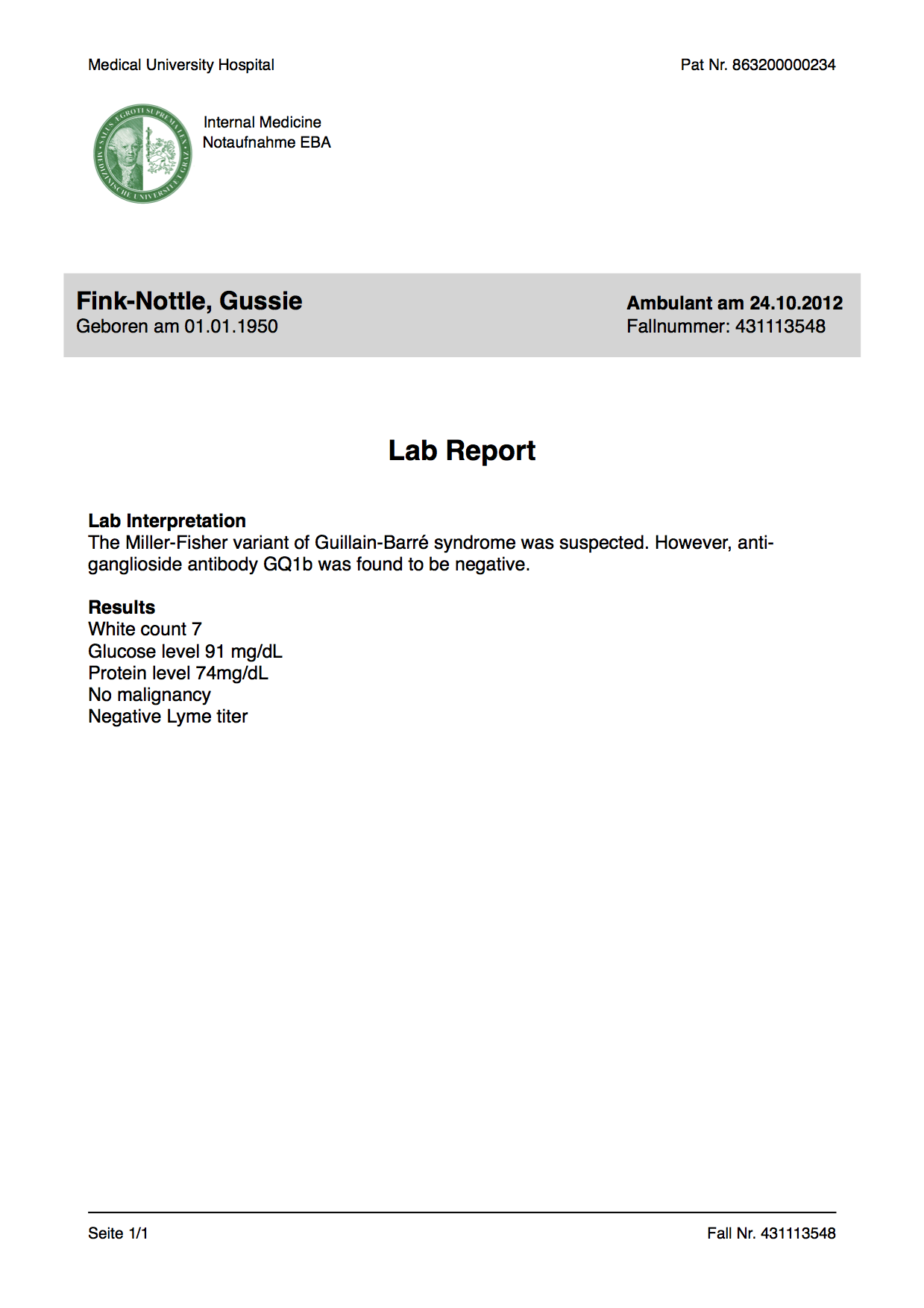

Supplement: Additional file 1 — Example case file. This file contains an example case in ZIP format that consists of 15 health records and a single JSON descriptor file (case.json) in the Casebook file format. The descriptor file describes the order of the health records, contains any annotations for each of the health records, and contains any questions that appear between health records. Portions of this case, including text, were extracted from a case published in the Journal of Medical Case Reports, an open-access journal where articles are made available under the terms of the Creative Commons Attribution Licence [27]. [file 1472-6947-14-66-S1.zip › 4.png]

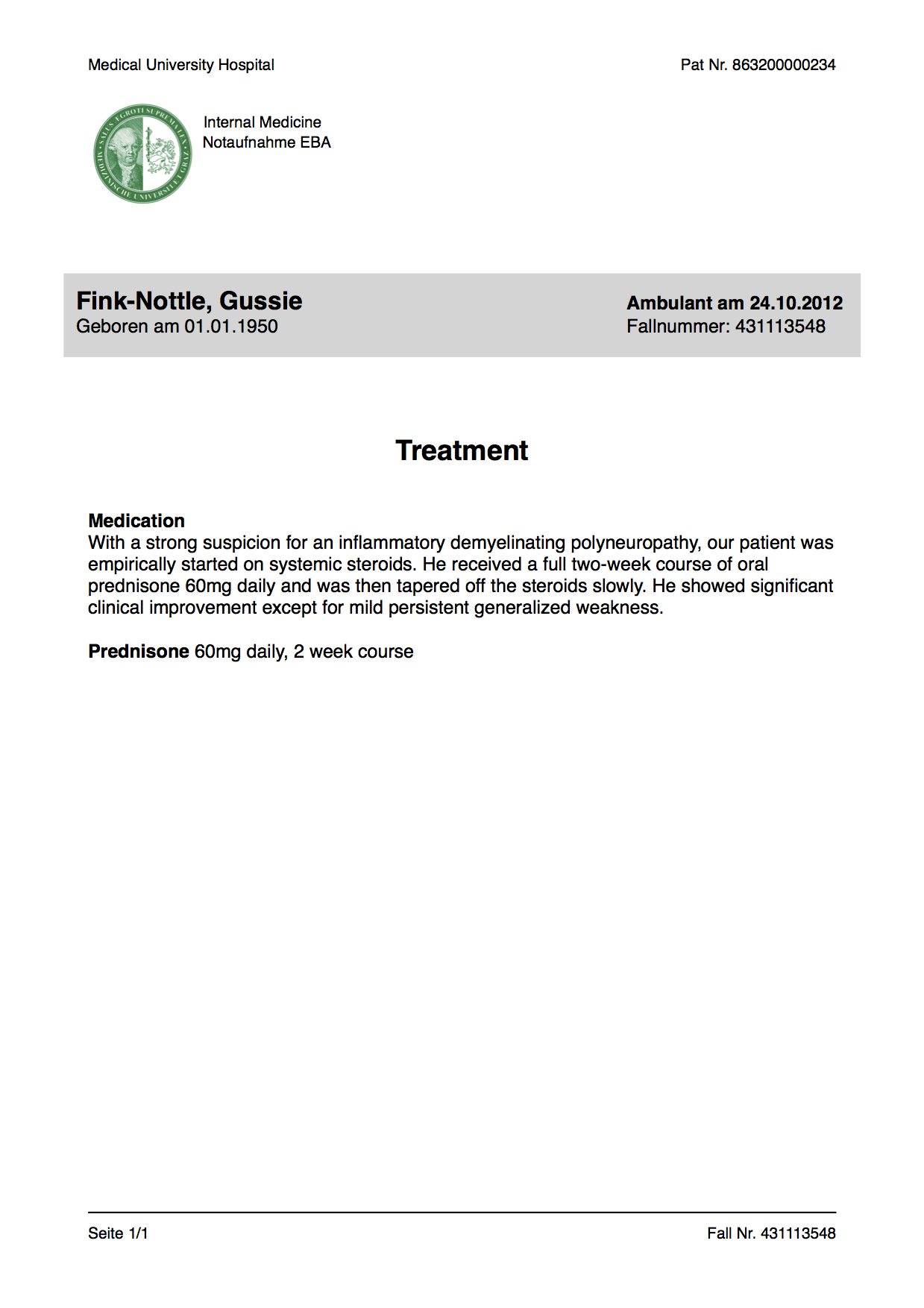

Supplement: Additional file 1 — Example case file. This file contains an example case in ZIP format that consists of 15 health records and a single JSON descriptor file (case.json) in the Casebook file format. The descriptor file describes the order of the health records, contains any annotations for each of the health records, and contains any questions that appear between health records. Portions of this case, including text, were extracted from a case published in the Journal of Medical Case Reports, an open-access journal where articles are made available under the terms of the Creative Commons Attribution Licence [27]. [file 1472-6947-14-66-S1.zip › 5.png]

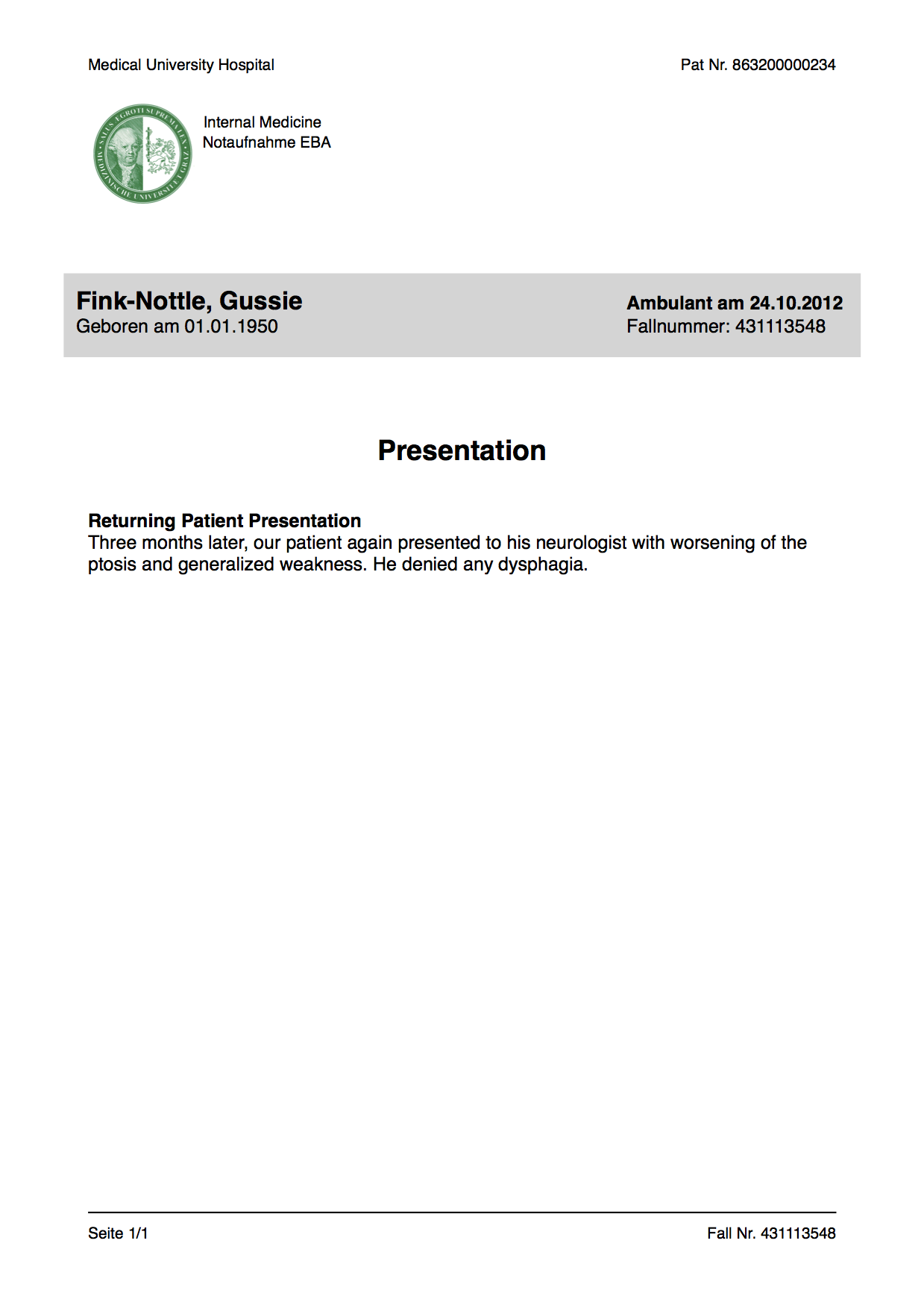

Supplement: Additional file 1 — Example case file. This file contains an example case in ZIP format that consists of 15 health records and a single JSON descriptor file (case.json) in the Casebook file format. The descriptor file describes the order of the health records, contains any annotations for each of the health records, and contains any questions that appear between health records. Portions of this case, including text, were extracted from a case published in the Journal of Medical Case Reports, an open-access journal where articles are made available under the terms of the Creative Commons Attribution Licence [27]. [file 1472-6947-14-66-S1.zip › 6.png]

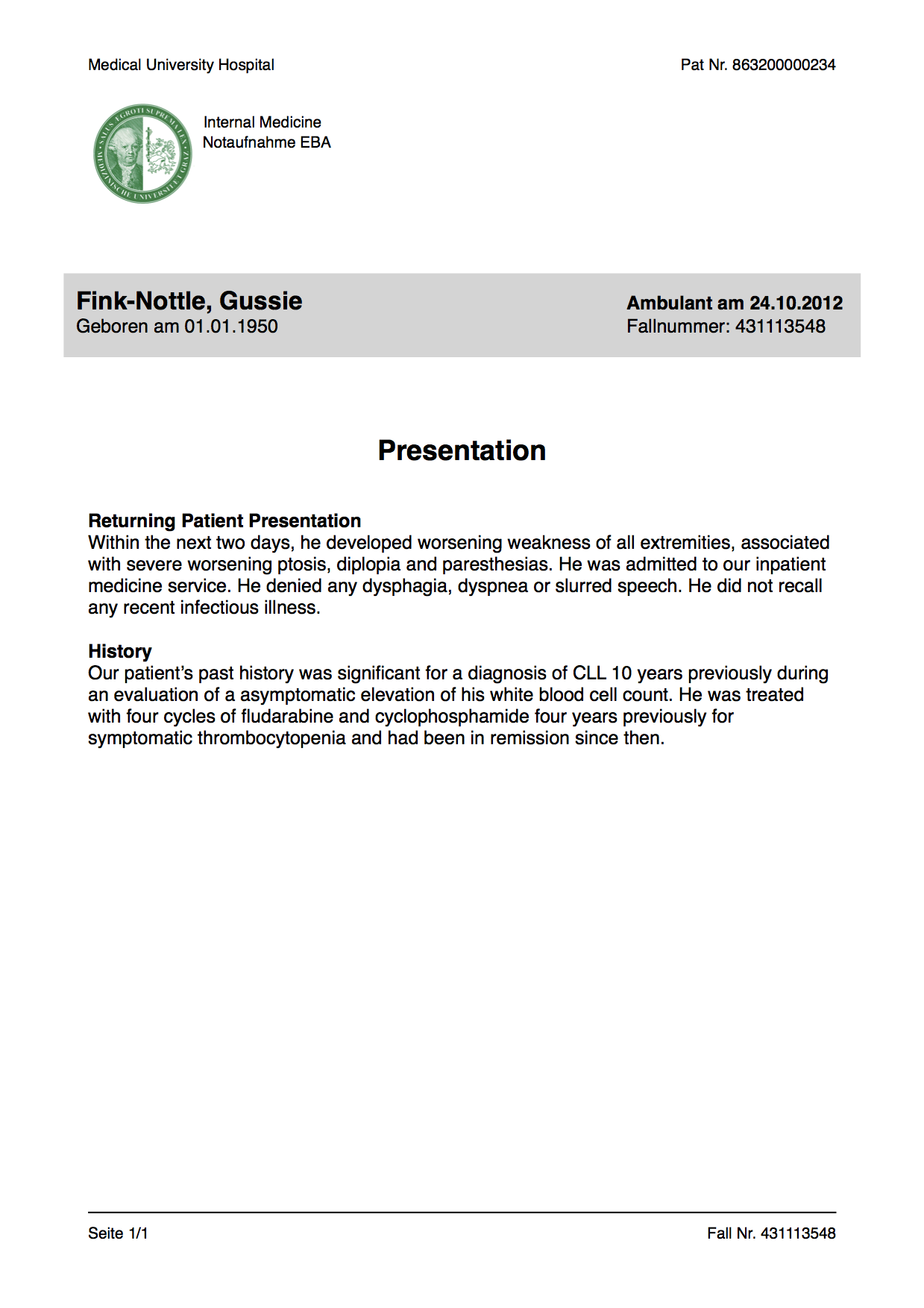

Supplement: Additional file 1 — Example case file. This file contains an example case in ZIP format that consists of 15 health records and a single JSON descriptor file (case.json) in the Casebook file format. The descriptor file describes the order of the health records, contains any annotations for each of the health records, and contains any questions that appear between health records. Portions of this case, including text, were extracted from a case published in the Journal of Medical Case Reports, an open-access journal where articles are made available under the terms of the Creative Commons Attribution Licence [27]. [file 1472-6947-14-66-S1.zip › 7.png]

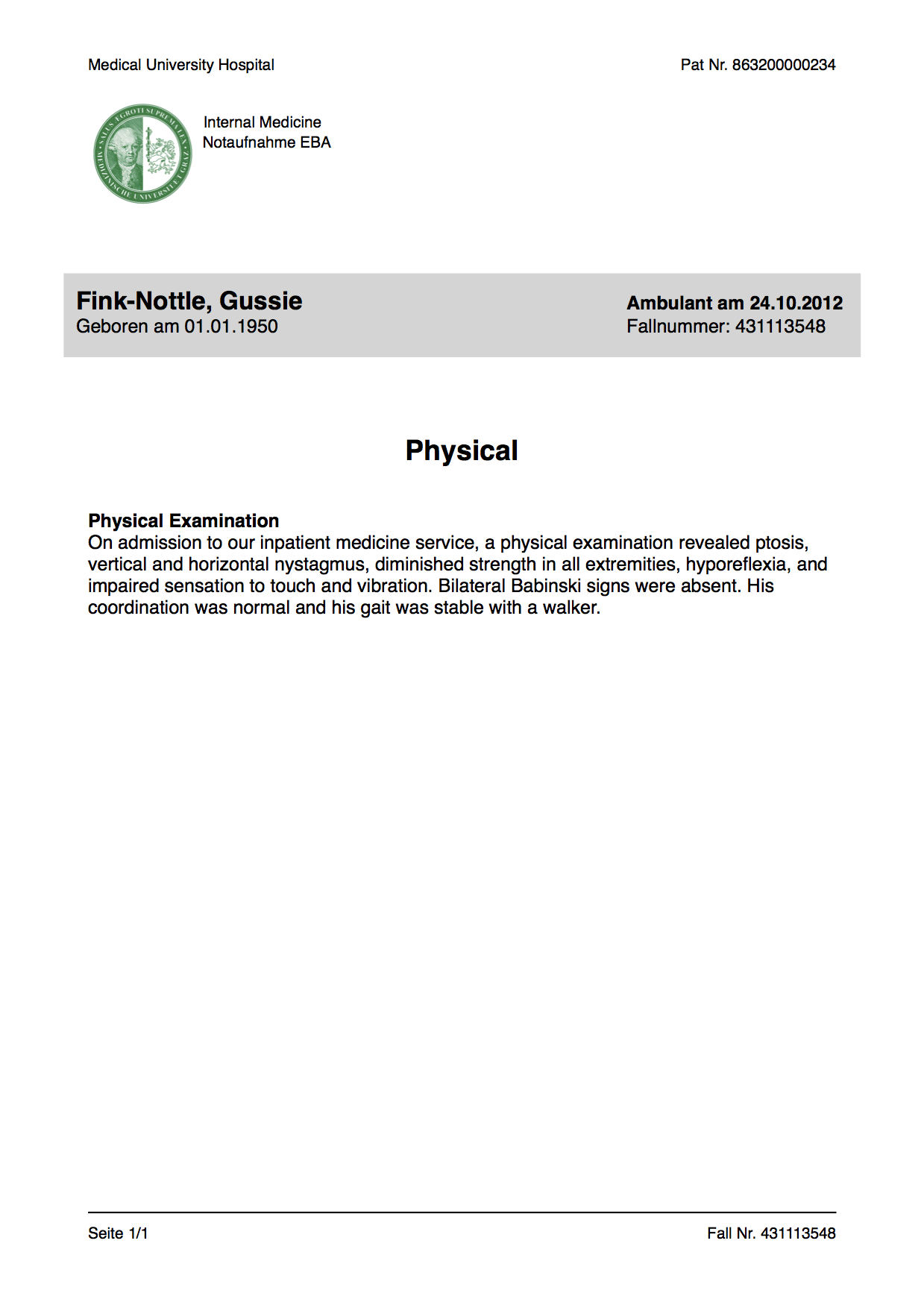

Supplement: Additional file 1 — Example case file. This file contains an example case in ZIP format that consists of 15 health records and a single JSON descriptor file (case.json) in the Casebook file format. The descriptor file describes the order of the health records, contains any annotations for each of the health records, and contains any questions that appear between health records. Portions of this case, including text, were extracted from a case published in the Journal of Medical Case Reports, an open-access journal where articles are made available under the terms of the Creative Commons Attribution Licence [27]. [file 1472-6947-14-66-S1.zip › 8.png]

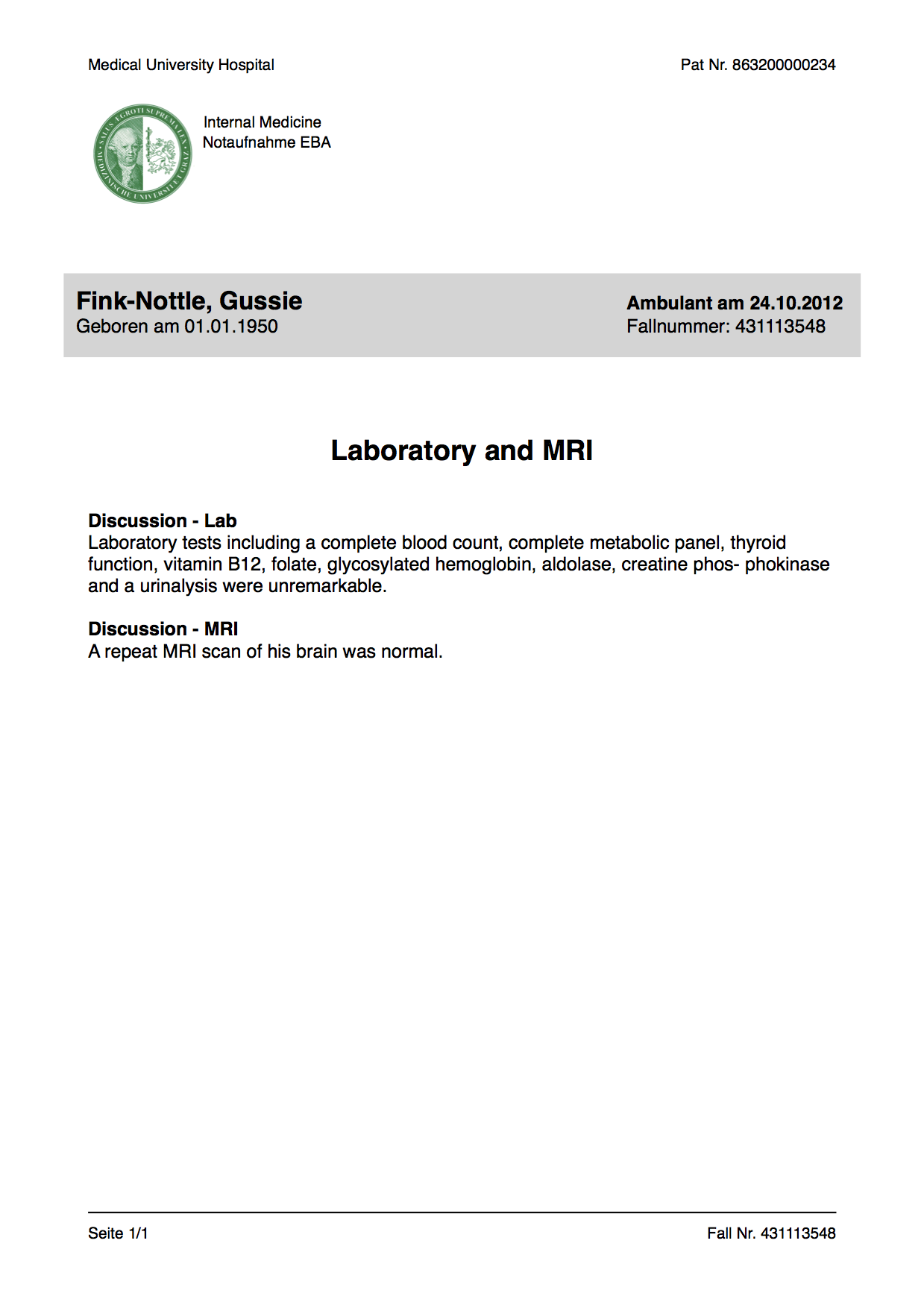

Supplement: Additional file 1 — Example case file. This file contains an example case in ZIP format that consists of 15 health records and a single JSON descriptor file (case.json) in the Casebook file format. The descriptor file describes the order of the health records, contains any annotations for each of the health records, and contains any questions that appear between health records. Portions of this case, including text, were extracted from a case published in the Journal of Medical Case Reports, an open-access journal where articles are made available under the terms of the Creative Commons Attribution Licence [27]. [file 1472-6947-14-66-S1.zip › 9.png]
