# Supplementary material for: Casebook: a virtual patient iPad application for teaching decision-making through the use of electronic health records
Source: BMC Med Inform Decis Mak. 2014 Aug 7;14:66. doi: 10.1186/1472-6947-14-66 (PMC4149039; doi:10.1186/1472-6947-14-66)
Supplement: Additional file 2 — Mini-website. HTML version of the example case that can be viewed using a browser. See Additional file 1 for this case in the Casebook file format. [file 1472-6947-14-66-S2.zip › index.htm]

JMCR Case


JMCR Case

# Case Description

Portions of this case, including text, were extracted from the following case report: Ammannagari N, Chikoti S, Bravin E: **Hodgkin's lymphoma presenting as a complex paraneoplastic neurological syndrome: a case report**. *Journal of Medical Case Reports* 2013, **7**:96.

# Visit 1

## 1. Presentation

|

### Annotation:

Stroke was considered as an early differential diagnosis.

### Question:

What is the correct medical term for a drooping eyelid?

1. Ptosis
2. Miosis
3. Mydriasis
4. Ectropion

---

## 2. Physical Examination

|

---

## 3. Consultation

|

### Question:

What statement regarding the procedure of a lumbar puncture is correct?

1. Important is a lying position, best with extended lumbar spine.
2. The spinal needle is inserted at L1/L2 heading 30° downwards.
3. The needle has to pass the ligamentum flavum and the dura mater together with the arachnoid membrane.
4. A blunt needle is needed to avoid spinal injuries.

---

## 4. Lab Report

|

---

## 5. Treatment

|

---

# Visit 2

## 6. Presentation

|

---

## 7. Further Presentation

|

---

## 8. Physical Examination

|

---

## 9. Laboratory and MRI

|

---

## 10. Examinations

|

---

## 11. Computed Tomography

|

---

## 12. CT Scan

|

### Annotation:

Computed tomography of the thorax showing the anterior superior mediastinal node measuring 1.7 by 1.3cm.

---

## 13. Biopsy

|

### Annotation:

Nodular sclerosing pattern of Hodgkin's lymphoma.

---

## 14. Treatment

|

---

## 15. Diagnosis

|

### Annotation:

Paraneoplastic neuropathies are seen in association with 4% to 5% of cancers. Hodgkin's lymphoma is also associated with multiple paraneoplastic neuropathies like cerebellar degeneration, acute inflammatory demyelinating polyneuropathy (Guillain-Barré), CIDP, chorea and ataxia, subacute sensory neuropathy, motor neuron disease, myasthenia gravis, stiff person syndrome and brachial neuropathy. The most common neurological syndrome described in the literature is subacute cortical cerebellar degeneration with more than 50 cases reported so far. Demyelinating neuropathies are very rare. Few cases of CLL associated with paraneoplastic demyelinating neuropathies like Guillain-Barré syndrome or Miller-Fisher syndrome are reported to date.

### Question:

Which classification is used to sort Hodgkin's lymphomas?

1. FIGO system
2. Dukes staging system
3. Gleason grading system
4. Whitmore-Jewett staging
5. Ann-Arbor staging

---


Ammannagari N, Chikoti S, Bravin E: **Hodgkin's lymphoma presenting as a complex paraneoplastic neurological syndrome: a case report**. *Journal of Medical Case Reports* 2013, **7**:96.
